# The Protective Effect of Quercetin on Endothelial Cells Injured by Hypoxia and Reoxygenation

**DOI:** 10.3389/fphar.2021.732874

**Published:** 2021-10-20

**Authors:** Meng-Ting Li, Jia Ke, Shu-Fen Guo, Yang Wu, Yue-Feng Bian, Li-Li Shan, Qian-Yun Liu, Ya-Jing Huo, Cen Guo, Ming-Yuan Liu, Ya-Jie Liu, Yan Han

**Affiliations:** ^1^ Department of Neurology, Yueyang Hospital of Integrated Traditional Chinese and Western Medicine, Shanghai University of Traditional Chinese Medicine, Shanghai, China; ^2^ Department of Neurology, Shenzhen Hospital, Southern Medical University, Shenzhen, China

**Keywords:** cerebral small vessel disease, endothelial cells, quercetin, oxidative stress, endoplasmic reticulum stress, blood brain barrier

## Abstract

**Background:** Cerebral small vessel disease (CSVD) is a group of clinical syndromes covering all pathological processes of small vessels in the brain, which can cause stroke and serious dementia. However, as the pathogenesis of CSVD is not clear, so the treatment is limited. Endothelial cell dysfunction is earlier than clinical symptoms, such as hypertension and leukosis. Therefore, the treatment of endothelial cells is expected to be a new breakthrough. Quercetin, a flavonoid present in a variety of plants, has the function of anti-inflammation and anti-oxidation. This study aimed to investigate the protective effect of quercetin on endothelial cell injury and provide a basic theory for subsequent application in the clinic.

**Methods:** Human brain microvascular endothelial cells (HBMECs) were cultured *in vitro*, and the injury model of endothelial cells was established by hypoxia and reoxygenation (H/R). The protective effects of quercetin on HBMECs were studied from the perspectives of cell viability, cell migration, angiogenesis and apoptosis. In order to further study the mechanism of quercetin, oxidative stress and endoplasmic reticulum stress were analyzed. What’s more, blood-brain barrier (BBB) integrity was also studied.

**Results:** Quercetin can promote the viability, migration and angiogenesis of HBMECs, and inhibit the apoptosis. In addition, quercetin can also activate Keap1/Nrf2 signaling pathway, reduce ATF6/GRP78 protein expression. Further study showed that quercetin could increase the expression of Claudin-5 and Zonula occludens-1.

**Conclusions:** Our experiments show that quercetin can protect HBMECs from H/R, which contains promoting cell proliferation, cell migration and angiogenesis, reducing mitochondrial membrane potential damage and inhibiting cell apoptosis. This may be related to its antioxidation and inhibition of endoplasmic reticulum stress. At the same time, quercetin can increase the level of BBB connexin, suggesting that quercetin can maintain BBB integrity.

## Introduction

Cerebral small vessel disease (CSVD) is an umbrella term that encompasses all pathological processes involving the small vessels in the brain and refers to a group of clinical, imaging, and pathological syndromes with various etiologies containing the intracranial arterioles to venules (diameter <400 μm) (Wardlaw et al., 2013; Wardlaw et al., 2019).The most common symptoms include mainly new onset subcortical small infarcts, lacunar foci of vascular origin, cerebral white matter hyperintensities, microbleeds, cerebral atrophy, and enlarged perivascular spaces (Wardlaw et al., 2013). With the progression of the disease, subclinical and early-stage patients can have emotional abnormalities, gait, memory, disorientation, and even stroke and dementia and other serious consequences. Up to 25% of stroke and 45% of dementia are caused by CSVD (Cannistraro et al., 2019), which brings a heavy socioeconomic burden and is a major problem that needs to be addressed by slow disease and health strategies.

Scholar has used dynamic contrast enhanced-MRI technique to find that blood-brain barrier (BBB) leakage is more prevalent in CSVD patients (Zhang et al., 2017). Extravasation of blood components may lead to local vascular changes and diffuse brain tissue damage. The BBB is a junction of endothelial cells, pericytes and astrocyte tight junctions (Keaney and Campbell, 2015). In addition, more and more scholars also believe that endothelial dysfunction plays a key role in the early development of CSVD (Hainsworth et al., 2015). Therefore, protecting endothelial cells may be a potential therapeutic strategy for CSVD.

Quercetin, a flavonoid present in several plants, such as *Polygonum cuspidatum* Sieb. et Zucc, has strong antioxidant and anti-inflammatory activities and can exert protective effects in various pathological conditions including cardiovascular disease, metabolic disorders, neurodegenerative diseases, diabetes, cancer and obesity (Dok-Go et al., 2003; Comalada et al., 2005; Cho et al., 2006). Pretreatment with quercetin significantly increased the expression levels of endogenous antioxidant enzymes in hippocampal CA1 pyramidal neurons of ischemia injured animals, showing strong antioxidant and neuroprotective effects (Chen et al., 2017). Recent studies have also found that quercetin has neuroprotective effects against ischemic injury while maintaining BBB integrity (Jin et al., 2019). However, its effect on brain microvascular endothelial cells (BMECs) under hypoxia and reoxygenation (H/R) injury is poorly studied, and the target protein of quercetin protecting BMECs has not been reported.

In our study, we explored the protective effect of quercetin on human brain microvascular endothelial cells (HBMECs) injured by H/R in culture. At the same time, we further studied the possible mechanism of its protective effect, so as to provide more theoretical basis for the clinical promotion of quercetin, and provide new ideas for the treatment of CSVD.

## Materials and Methods

### Media, Reagents and Antibodies

Quercetin (purity>98%) was acquired from Best Biological Technology Co., Ltd. (Chengdu, China). HBMECs were purchased from Qingqi Biotechnology Development Co., Ltd (Shanghai, China). Fetal bovine serum (FBS) was obtained from Biological Industries (Kibbutz Beit Haemek, Israel). Penicillin/streptomycin and dulbecco’s modified eagle medium (DMEM) were purchased from Hyclone (GA, United States ). Mitochondrial membrane potential (JC-1) test kit, BCA protein test kit, 2,7-dichlorodi-hydrofluorescein diacetate (DCFH-DA) and NP40 lysate were purchased from Beyotime Biotechnology (Shanghai, China). Phosphatase preparation and Complete protease inhibitor were purchased from Roche (Shanghai, China). Apoptosis Kit was purchased from BD Biosciences (Sparks, MD, United States ). Malondialdehyde (MDA), Superoxide dismutase (SOD), Intercellular cell adhesion molecule-1 (ICAM-1), Vascular cell adhesion molecule-1 (VCAM-1) enzyme linked immunosorbent assay (ELISA) was purchased from Abcam company (Cambridge, United Kingdom). Antibodies against Nuclear factor E2-related factor 2 (Nrf2), Kelch Like ECH Associated Protein 1 (Keap1), Activating transcription factor 6 (ATF6), Glucose-regulated protein 78 (GRP78), Zonula occludens 1 (ZO-1), Claudin-5, GAPDH was purchased from Cell Signaling Technology (Danvers, United States). Horseradish peroxidase (HRP)-conjugated anti-rabbit IgG was obtained from Jackson company (Pennsylvania, United States). SDS-PAGE rapid dispensing kit and ECL chromogenic solution were purchased from EpiZyme Biotechnology (Shanghai, China).

### Cell Culture and Injury Model

HBMECs were cultured in DMEM containing 10% FBS and 1% penicillin-streptomycin, at 37°C incubator with 5% CO_2_. To cause endothelial cell damage, HBMECs were treated with 12 h hypoxia followed by 8 h reoxygenation, and the specific procedures were as follows (Warpsinski et al., 2020): HBMECs were first incubated with serum-free medium in a hypoxia incubator (1% O_2_, 5% CO_2_, 94% N_2_) for 12 h, and then the medium was changed to normal medium containing 10% FBS, while they were incubated in a normoxia incubator (95% O_2_, 5% CO_2_) for 8 h.

### Quercetin Treatment

2 mg Quercetin was dissolved in 331 μL DMSO to form a stock solution with a concentration of 1 mmol/L and stored at −20°C. The working solution was diluted to 0.1, 0.5, 1, 2, 5, 10 μmol/L with DMEM, in which the percentage of DMSO was below 0.1%. Once cells had been adherent, quercetin with serum-free medium was added, and the plates were subsequently placed into the hypoxia incubator. After 12 h, cells were removed, changed to quercetin with serum medium, and placed in a normoxia incubator for 8 h.

### Cell Viability Assay

The effects of quercetin on the viability of HBMECs were examined by Cell Counting Kit-8 according to the operator’s manual. The safe doses of quercetin to the cells in the absence of damage to the endothelial cells were first assessed, and then the protective effects of it in the condition of cell damage were tested. All experimental results were repeated at least three times.

### Scratch Healing and Tube Formation Assay

Migration experiments were performed using a cell scratch method. Before quercetin treatment, a straight perpendicular line was drawn at the bottom of the culture plate and photographed for recording for 0 h, and the cells were subsequently subjected to quercetin and modeling treatment before taking photographs for recording. The anterior and posterior areas were contrasted twice.

Tube formation mimicking cell angiogenic ability, the Matrigel (9–12 mg/ml) was placed on the bottom of the culture plate, then quercetin and modeling treated cells were plated on top, after 8 h, pictures were taken. The branching generation of blood vessels was analyzed and compared using ImageJ.

### Mitochondrial Membrane Potentials and Apoptosis Assay

The mitochondrial membrane potential change and apoptosis kit were used to detect cell apoptosis. The alteration of mitochondrial membrane potential was detected by staining HBMECs with JC-1 fluorescent probe. JC-1 (1 ×) staining working solution was added and incubated in a 37°C incubator for 20 min before being washed twice with JC-1 (1 ×) staining buffer and finally added to each well for PBS. Then, the high content cell imager was used to analysis.

HBMECs were collected from the 6-well plates, centrifuged to remove the supernatant, subsequently add 1 × Annexin V binding solution to blow and mix the cells, then add FITC Annexin V dye to incubate in the dark at room temperature for 10 min, add propidium iodide (PI) staining solution 5 min before the machine, and Beckman flow cytometer was employed to detect.

### ELISA

After the cells were treated with H/R or quercetin, the supernatant was collected. ICAM-1, VCAM-1, SOD and MDA were detected using ELISA kits according to the operator’s manual.

### Reactive Oxygen Species (ROS) Assay

The ability of ROS generation was detected by the DCFH-DA probe, which was evaluated by the high content cell imager, and analyzed by fluorescence using ImageJ.

### Western Blotting

HBMECs were collected from culture plates, NP40 lysate adding phosphatase preparation and complete protease inhibitor was used to extract proteins from cells. Protein content was determined and 30 μg protein was taken for immunoblotting experiments. SDS-PAGE gel was used for electrophoresis, blocked with 5% nonfat dry milk after electrotransfer to PVDF membrane for 1 h at room temperature. Then anti-Nrf2 (rabbit, 1:1,000), anti-Keap1 (rabbit, 1:1,000), anti-ATF6 (rabbit,1: 1,000), anti-GRP78 (rabbit, 1:1,000), anti-ZO-1 (rabbit, 1:1,000), anti-Claudin-5 (rabbit, 1:1,000) antibodies were incubated overnight at 4°C. After washing the next day, diluted Goat anti rabbit secondary antibodies (IgG HRP, 1:10,000) were added and incubated for 1 h. Finally, detection was developed after washing three times in TBST.

### Proteomic Analysis

HBMECs were treated with H/R in the present of quercetin (1 μmol/L). Subsequently, the cells were gently scraped from the culture plate with a cell scraper, collected in cryovials, quickly placed in liquid nitrogen, and then stored at −80°C for 5 min iTRAQ technology was used to do the proteomic analysis, the proteins with *p* value <0.05 and ratio multiple change >1.2 or <0.83 were defined as differentially expressed proteins.

### Statistical Analysis

Data were fit with normal distribution, and mean ± standard deviation was used to express the data. The GraphPad Prism 8 was used for statistical analysis. Two independent samples were analyzed by *t*-test, and the measurement data were described by mean ± Standard deviation (SD). The comparison between groups was conducted by one-way analysis of variance (ANOVA). *p* value <0.05 indicated that the difference was statistically significant.

## Results

### Results

#### Effect of Quercetin on Cell Viability

**FIGURE 1:**
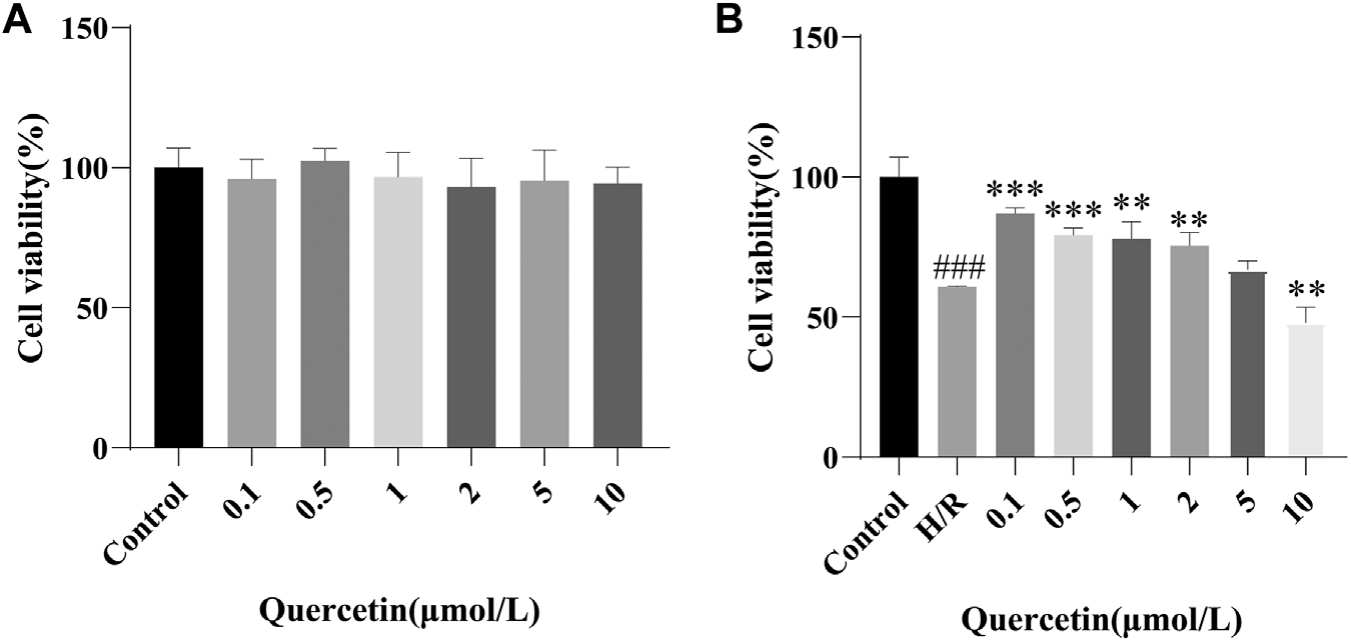
(A) HBMECs were treated with quercetin (0, 0.1, 0.5, 1, 2, 5, 10 μmol/L) for 24 h, viability was measured using CCK-8 method (B) HBMECs were treated with quercetin (0, 0.1, 0.5, 1, 2, 5, 10 μmol/L) and H/R treatment, viability was measured using CCK-8 method. ^###^ *p* < 0.001 compared to control group; ^**^ *p* < 0.01, ^***^ *p* < 0.001 compared to H/R group. H/R: hypoxia and reoxygenation, n = 3.

To test the effect of quercetin on cell viability, we first examined a safe dose of quercetin on the cells, as shown in Figure 1A, which had no impact for cell viability at 0.1–10 μmol/L. Next, for searching for an effective dose on the basis of endothelial cell damage, we showed that quercetin at 0.1–1 μmol/L could promote cell viability in H/R-HBMECs, as shown in Figure 1B. Subsequent experiments were carried out around this concentration range. It is worth noting that when the drug concentration reached 10 μmol/L, cytotoxicity appeared. We thought that when HBMECs were damaged by H/R, its tolerance decreases. Therefore, when the drug concentration was slightly higher, the damage to cells increased. Therefore, cytotoxicity occurred when the drug concentration is 10 μmol/L.

#### Effect of Quercetin on Cell Migration and Angiogenesis

**FIGURE 2:**
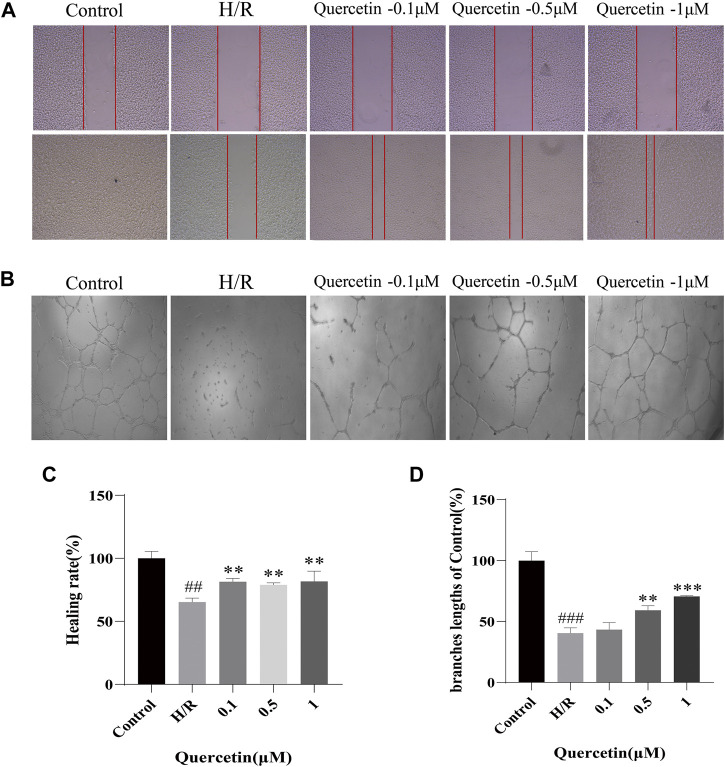
**(A)** The distance between scratches in the presence of quercetin (0.1, 0.5, 1 μmol/L) was measured using scratch method **(B)** The branches length of the tube under the different concentrations of quercetin (0.1, 0.5, 1 μmol/L) was detected with tube formation method **(C)** Healing rate was calculated **(D)** Branches length data was displayed. ^##^ *p* < 0.01, ^###^ *p* < 0.001 compared to control group; ^**^ *p* < 0.01, ^***^ *p* < 0.001 compared to H/R group. μM: μmol/L; H/R: hypoxia and reoxygenation, n = 3.

To examine the effects of quercetin on the migratory and angiogenic capacities of endothelial cells, HBMECs were subjected to H/R treatment while quercetin was administered. We performed a scratch assay to test the cell migration ability, which showed that H/R treatment weakened the migration ability of H/R-HBMECs compared with the control, and quercetin could significantly promote cell migration at a concentration of 0.1–1 μmol/L. Tubulogenesis was used to mimic *in vitro* angiogenesis, and its branch length represents angiogenic capacity. The results showed that H/R treatment resulted in reduced angiogenic capacity, but quercetin 0.1–1 μmol/L could reverse this injury, as shown in Figure 2.

#### Effect of Quercetin on Cell Apoptosis

**FIGURE 3:**
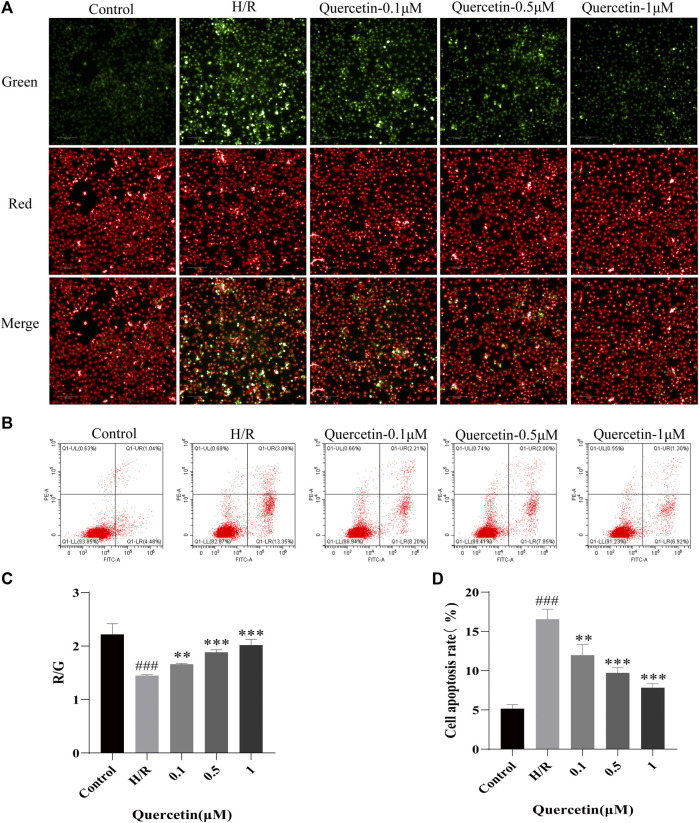
**(A)** The mitochondrial membrane was examined using JC-1 probe **(B)** Apoptosis was evaluated using flow cytometry **(C)** R/G represents altered mitochondrial membrane potential **(D)** Apoptosis data was analyzed. ^###^ *p* < 0.001 compared to control group; ^**^ *p* < 0.01, ^***^ *p* < 0.001 compared to H/R group. μM: μmol/L; R/G: Red/Green; H/R: hypoxia and reoxygenation, n = 3.

To test the potential of quercetin to inhibit endothelial cell apoptosis, we employed mitochondrial membrane potential and apoptosis for evaluation. When cells were injured to undergo apoptotic events, the mitochondrial membrane potential was reduced, cytoplasmic red fluorescence was significantly reduced, green fluorescence was increased, and Red/Green (R/G) was finally adopted to represent the alteration of mitochondrial membrane potential, which will decrease when the mitochondrial membrane potential is compromised. Our results showed that after H/R treatment, the mitochondrial membrane potential of the cells decreased, as indicated by increased green fluorescence. With increasing doses of quercetin, red fluorescence was constantly enhanced, indicating that quercetin can dose dependently elevate mitochondrial membrane potential (Figure 3A, C). Further, Flow cytometry was employed to directly examine cell apoptosis. The results showed that HBMECs exhibited an elevated rate of apoptosis after H/R treatment, whereas quercetin at 0.1–1 μmol/L exhibited a clear ability to inhibit apoptosis (Figure 3B, D).

#### Effect of Quercetin on Cell Adhesion

**FIGURE 4:**
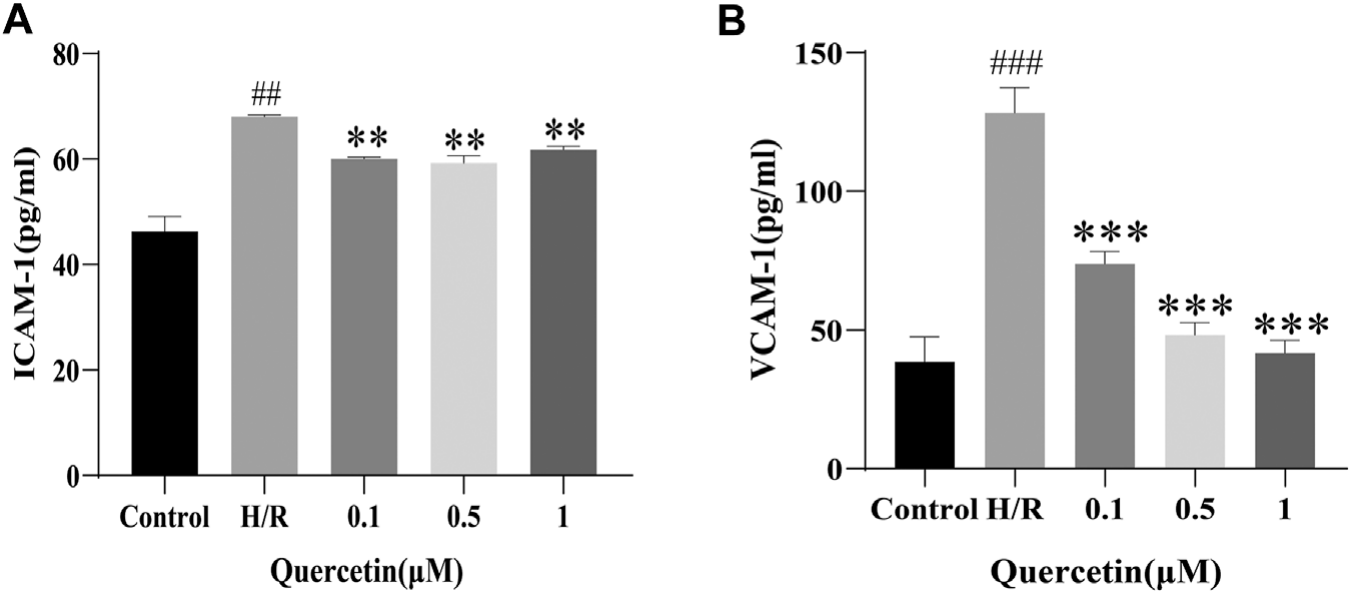
**(A)** ICAM-1 was detected with ELISA **(B)** VCAM-1 was evaluated using ELISA. ^##^ *p* < 0.01, ^###^ *p* < 0.001 compared to control group; ^**^ *p* < 0.01, ^***^ *p* < 0.001 compared to H/R group. μM: μmol/L; ICAM-1: Intercellular cell adhesion molecule-1; VCAM-1: Vascular cell adhesion molecule-1; H/R: Hypoxia and reoxygenation, n = 3.

Once endothelial cells are damaged, some adhesion molecules, such as ICAM-1 and VCAM-1 are produced, adsorbing toxic substances into the BBB, and damaging the brain parenchyma (Hauptmann et al., 2020). To examine the effect of quercetin on the ability of H/R-HBMECs to produce adhesion molecules, ICAM-1 and VCAM-1 were measured using ELISA. In our study, H/R resulted in increased levels of ICAM-1 and VCAM-1, but the addition of quercetin under the concentration of 0.1–1 μmol/L decreased this effect (Figure 4).

#### Quercetin Could Inhibit Oxidative Stress

**FIGURE 5:**
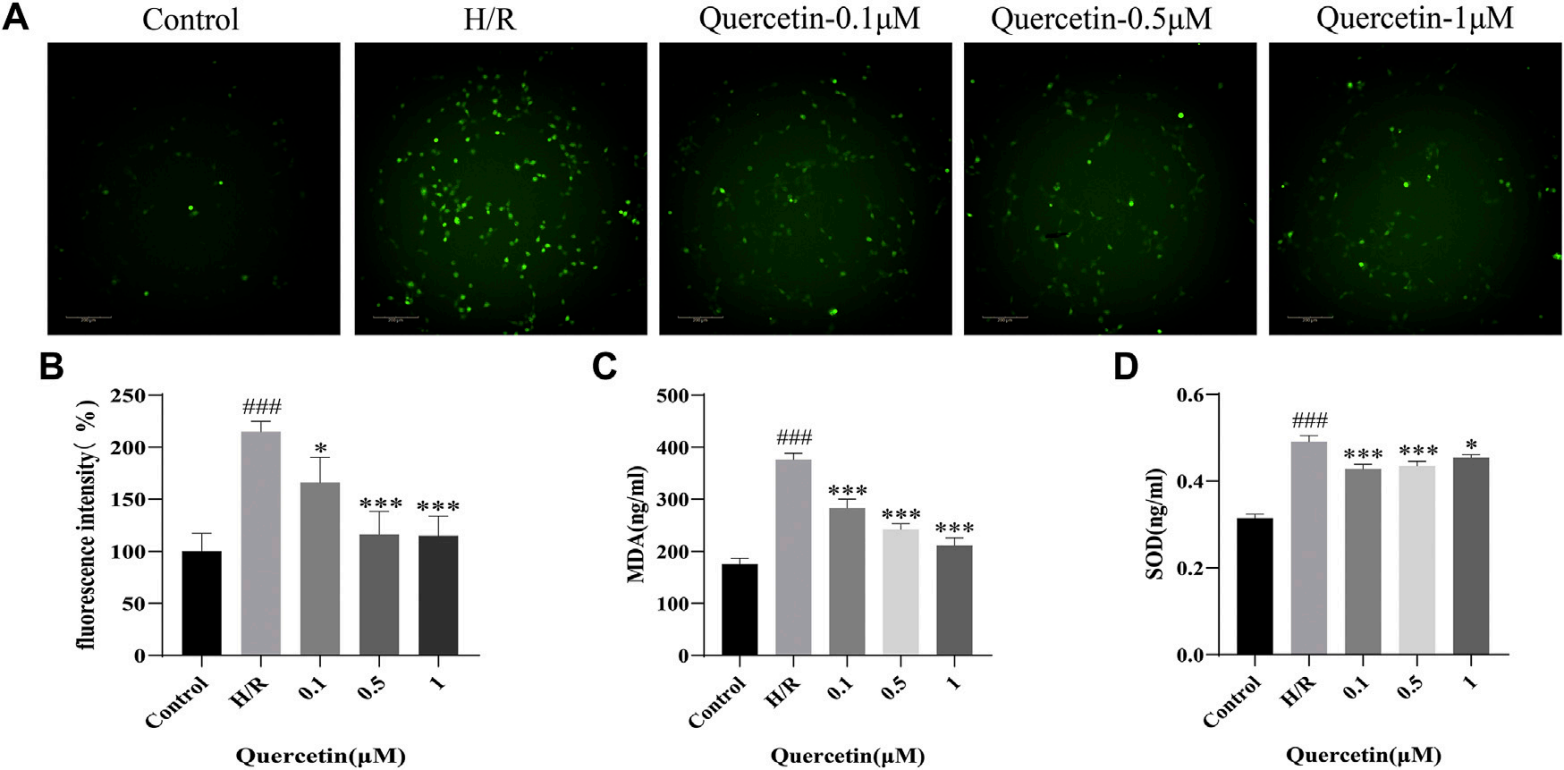
**(A)** HBMECs was treated with quercetin (0.1, 0.5, 1 μmol/L) and H/R, ROS level was measured using DCFH-DA probe **(B)** Fluorescence intensity represents ROS expression **(C)** MDA was detected with ELISA **(D)** SOD was evaluated using ELISA. ^###^ *p* < 0.001 compared to control group; ^*^ *p* < 0.05, ^***^ *p* < 0.001 compared to H/R group. μM: μmol/L; ROS: Reactive oxygen species; MDA: Malondialdehyde; SOD: Superoxide dismutase; H/R: Hypoxia and reoxygenation, n = 3.

Quercetin is a flavonoid with strong antioxidant capacity, so we employed DCFH-DA probe to detect ROS. We observed increased ROS generation in HBMECs exposed to H/R, which was markedly reduced by quercetin treatment at the concentrations of 0.1–1 μmol/L. Meanwhile, we detected the level changes of oxidative stress products SOD and MDA with ELISA, and the results showed that quercetin at the concentration of 0.1–1 μmol/L was able to decrease SOD and MDA, which were elevated by H/R, thus indicating that quercetin could reduce the damage of HBMECs from oxidative stress (Figure 5).

#### Quercetin Could Regulate Keap1/Nrf2 and ATF6/GRP78 Proteins

**FIGURE 6:**
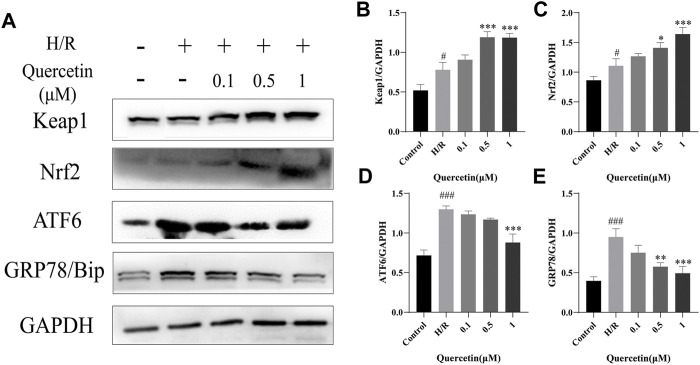
**(A)** Protein expressions were evaluated with Western blotting **(B)** Keap1 quantification of Western blotting result was calculated **(C)** Nrf2 expression quantification of Western blotting result was inhibited **(D)** ATF6 expression data was analyzed **(E)** GRP78 expression data was analyzed. ^#^ *p* < 0.05, ^###^ *p* < 0.001 compared to control group; ^*^ *p* < 0.05, ^**^ *p* < 0.01, ^***^ *p* < 0.001 compared to H/R group. μM: μmol/L; Keap1: Kelch Like ECH Associated Protein 1; Nrf2: Nuclear factor E2-related factor 2; ATF6: Activating transcription factor 6; GRP78: Glucose-regulated protein 78; H/R: Hypoxia and reoxygenation, n = 3.

To further explore the protective mechanism of quercetin on HBMECs, we analyzed the expression levels of oxidative stress and endoplasmic reticulum stress-related proteins. Keap1/Nrf2 is known to regulate antioxidant responses *in vivo*. Oxidative stress injury forces endothelial cells to undergo a stress response, resulting in increased Keap1/Nrf2 levels (Warpsinski et al., 2020). In the present study, H/R resulted in the activation of cellular antioxidant response mechanisms and increased levels of Keap1 and Nrf2, which could be further strengthened by quercetin at 0.5–1 μmol/L to enhance the antioxidant capacity of HBMECs. ATF6/GRP78 is one of the pathways that regulate endoplasmic reticulum (ER) homeostasis. ER stress can aggravate endothelial cell injury (Nie et al., 2020). In our study, ER stress was activated by H/R, and the levels of ATF6 and GRP78 were increased. Quercetin at 1 μmol/L was able to significantly reduce the protein levels of both, inhibit ER stress, and protect HBMECs from H/R injury (Figure 6).

#### Quercetin Could Maintain BBB Integrity

**FIGURE 7:**
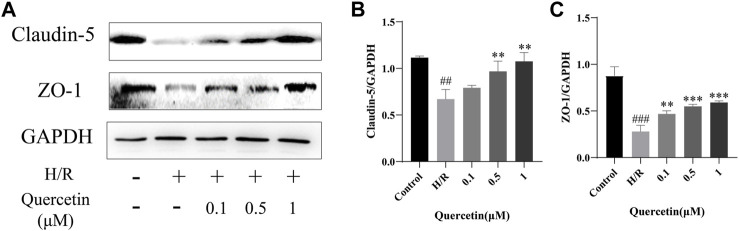
**(A)** Protein expressions were evaluated with Western blotting **(B)** Claudin-5 quantification of Western blotting result was calculated **(C)** ZO-1 expression quantification of Western blotting result was inhibited. ^##^ *p* < 0.01, ^###^ *p* < 0.001 compared to control group; ^**^ *p* < 0.01, ^***^ *p* < 0.001 compared to H/R group. μM: μmol/L; ZO-1: Zonula occludens 1; H/R: Hypoxia and reoxygenation, n = 3.

BMECs are the backbone of BBB structure, and their death or apoptosis after H/R injury affects BBB integrity and functionality (Ding et al., 2019). In this paper, the function of endothelial cells was reflected by the detection of changes in the expression of BBB associated proteins ZO-1 and Claudin-5. Compared with the control group, the expression of ZO-1 and Claudin-5 was decreased in H/R injury, indicating that the BBB was damaged and this damage could be reversed by quercetin at the concentrations of 0.5–1 μmol/L, indicating that quercetin can maintain the function of endothelial cells (Figure 7).

#### Proteomic Analysis

**FIGURE 8:**
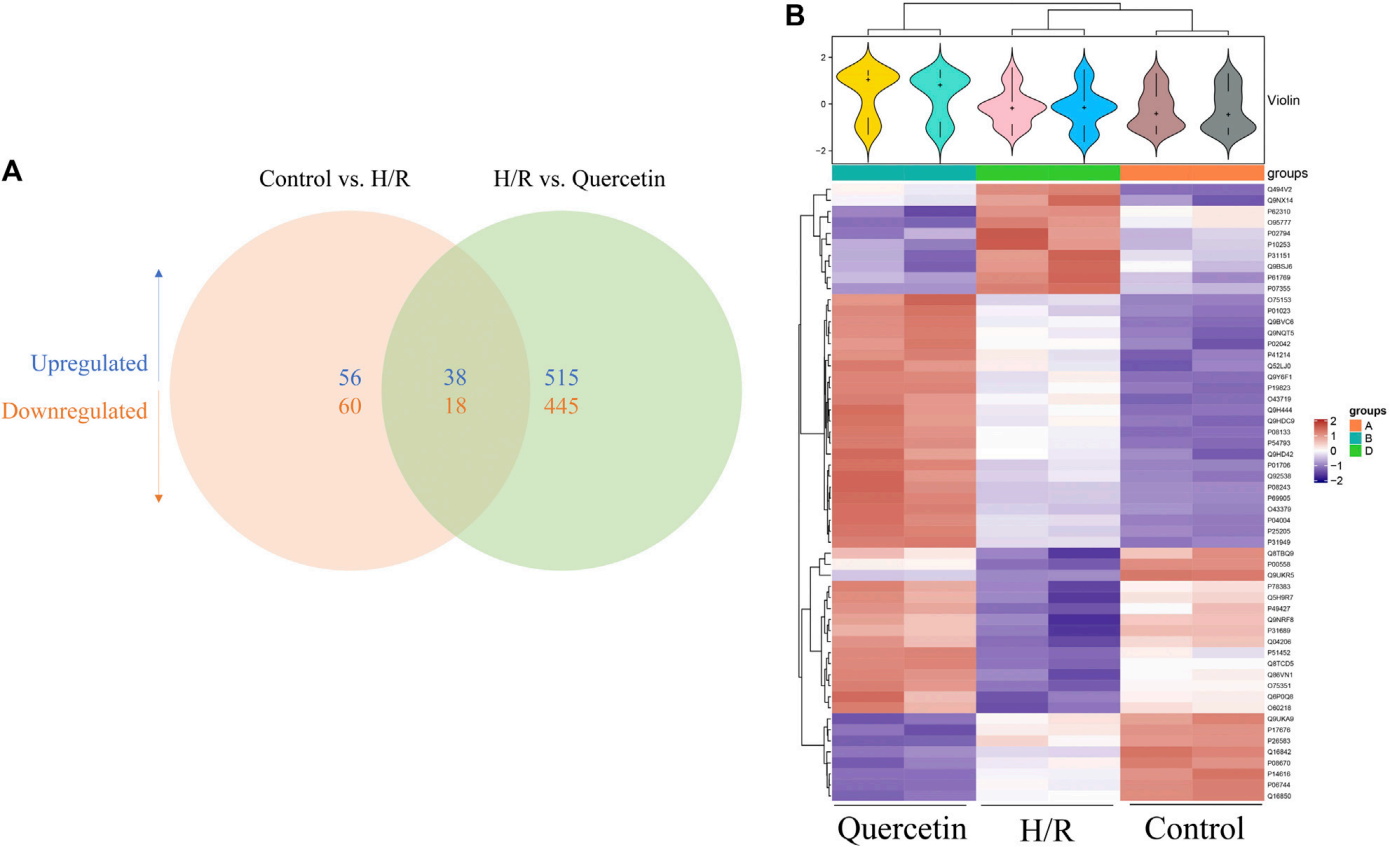
**(A)** Venn diagram of the distribution in each comparison group **(B)** Heatmap of the 56 common differentially expressed proteins. H/R: Hypoxia and reoxygenation.

To find the differentially expression proteins (DEPs) of H/R injured HBMECs in the presence of quercetin or not, iTRAQ was used to conduct the proteomics analysis. Proteins exhibiting a *p* < 0.05 and a ratio fold change >1.2 or <0.83 were defined as DEPs. In our study, the differences in protein among the control, H/R, and quercetin groups were compared. The results showed that 172 proteins were identified as DEPs between the control and H/R groups, among which 94 were upregulated and 78 were downregulated in the H/R group. There were 1,016 proteins identified as DEPs between H/R group and the quercetin group, of which 553 were up-regulated and 463 were down-regulated in the quercetin group, as shown in Figure 8A. Between control vs H/R and H/R vs quercetin group, 56 of the same DEPs were shared between the two groups. The expressions of these DEPs were analyzed by hierarchical clustering as shown in Figure 8B. Among these DEPs, top 20 were shown in Table1. Among these 20 commons, insulin receptor-related protein (INSRR), dual specificity protein phosphatase 3 (DUSP3), annexin A2 (ANXA2), hemoglobin subunit alpha (HBA1), phosphoglycerate kinase 1 (PGK1), vitronectin (VTN), glucose-6-phosphate isomerase (GPI) are related to endothelial cells.

**TABLE 1:** Top 20 of the common differentially expressed proteins.

Accession	Gene name	Description
P14616	INSRR	Insulin receptor-related protein
P31949	S100A11	Protein S100-A11
P51452	DUSP3	Dual specificity protein phosphatase 3
Q8TCD5	NT5C	5′ (3′)-deoxyribonucleotidase, cytosolic type
P07355	ANXA2	Annexin A2
O95777	LSM8	U6 snRNA-associated Sm-like protein LSm8
P25205	MCM3	DNA replication licensing factor MCM3
O43379	WDR62	WD repeat-containing protein 62
P49427	CDC34	Ubiquitin-conjugating enzyme E2 R1
Q9UKR5	ERG28	Ergosterol biosynthetic protein 28
P69905	HBA1	Hemoglobin subunit alpha
P00558	PGK1	Phosphoglycerate kinase 1
O75351	VPS4B	Vacuolar protein sorting-associated protein 4B
P54793	ARSF	Arylsulfatase F
Q16850	CYP51A1	Lanosterol 14-alpha demethylase
Q9BVC6	TMEM109	Transmembrane protein 109
P04004	VTN	Vitronectin
P06744	GPI	Glucose-6-phosphate isomerase
P01706	IGLV2-11	Immunoglobulin lambda variable 2–11
P19823	ITIH2	Inter-alpha-trypsin inhibitor heavy chain H2

## Discussion

CSVD, as an increasing medical and socioeconomic burden, has rapidly attracted attention. But surprisingly, the pathogenesis of CSVD remains obscure at present, rendering no clear scheme for its treatment either. Given that the later stages of CSVD can progress to severe outcomes such as stroke and dementia, targeting the early lesions for treatment can effectively delay the progression of CSVD (Cannistraro et al., 2019). It has been shown that the early pathological changes of CSVD lie in endothelial cell dysfunction and drugs that stabilize its dysfunction may improve the vulnerability of cerebral white matter in CSVD lesions (Rajani et al., 2018). Given the role played by endothelial cells in CSVD, we set out to investigate the protective effects of traditional Chinese medicine on endothelial cells using HBMECs.

Quercetin is a flavonoid present in a variety of plants (Nawrot-Hadzik et al., 2019). It has strong antioxidant and anti-inflammatory activities and can exert protective effects in various pathological conditions. In this study, we demonstrated that quercetin can ameliorate H/R injury, and several experimental data confirm our conclusion that quercetin can promote cell viability, cell migration, angiogenesis, increase mitochondrial membrane potential and inhibit apoptosis.

Considering that quercetin has strong antioxidant activity, we investigated the mechanism by which quercetin protects HBMECs in terms of antioxidation. ROS levels can be significantly increased by oxidative stress (Wu et al., 2018), and the accumulation of ROS can cause many diseases, including cardiovascular diseases, endothelial dysfunction and aging related diseases, and neurodegenerative diseases (Chen et al., 2015; Jakaria et al., 2018; Santos et al., 2018). Moreover, oxidative stress occurs lipid peroxidation, producing MDA, which will destroy the body’s oxidative antioxidant balance and increase endothelial cell damage (Yang et al., 2021). Keap1/Nrf2 is considered to be the most important self-anti-oxidative stress pathway at present, and activation of this pathway can significantly improve endothelial cell dysfunction. In particular, ROS help promote the activation of the Nrf2 signaling pathway (Selimoglu-Buet et al., 2017). We have demonstrated that quercetin can decrease ROS and MDA generation, while increasing Keap1 and Nrf2 protein expression, suggesting that quercetin may attenuate endothelial cell injury by decreasing oxidative stress responses via activating the Keap1/Nrf2 signaling pathway.

Not only that, sustained ER stress activates the unfolded protein response and alters the expression of antioxidant genes, leading to endothelial cell apoptosis (Tang et al., 2019). Moreover, oxidative stress can also cause the disturbance of protein folding in the ER, provoke ER stress, and aggravate endothelial cell injury (Hetz, 2012). ER stress can be activated by these three pathways: PKR-like ER kinase (PERK), inositol requiring enzyme 1 (IRE1), and the activating ATF-6, which protect cells from ER stress under homeostatic conditions (Hetz et al., 2020). But when affected by external adverse stimuli, these three pathways would activate the ER stress response and induce cell apoptosis. Moreover, studies have shown that inhibition of the ER stress response can reverse H/R-induced endothelial cell dysfunction (Chen et al., 2020).In our study, quercetin could reduce the elevated ATF6/GRP78 content caused by H/R, inhibit the endoplasmic reticulum stress response, and protect endothelial cells.

As a major component of the BBB, the functions of endothelial cells include maintaining its integrity. BBB dysfunction causes leakage of fluids, proteins, and other plasma components into perivascular tissues, further impairing cerebral vasodilation and nutrient transport (Wardlaw et al., 2019). When endothelial cells are damaged and the integrity of BBB will be destroyed, so protecting endothelial cells can further maintain the integrity of BBB.Claudin-5 is highly expressed in BMECs and is involved in constituting the backbone of tight junction chains to regulate BBB permeability (Yang et al., 2020). Endothelial cells are anchored to the actin cytoskeleton by scaffolding proteins such as ZO-1, rendering tight junctions between endothelial cells that maintain the tightness of the BBB (Zhang et al., 2020). In our study, the expression of claudin-5 and ZO-1 was used to represent BBB integrity. Our results showed that the protein levels of claudin-5 and ZO-1 were decreased in HBMECs subjected to H/R injury, indicating that BBB integrity was disrupted, and quercetin could reduce this injury by elevating claudin-5 and ZO-1 protein levels.

Furthermore, to find the potential targets of quercetin on HBMECs, i-TRAQ was labeled into cells. After proteomic analysis, top 20 of the common differentially expressed proteins were investigated. Among them, INSRR, DUSP3, ANXA2, HBA1, PGK1, VTN, GPI are related to endothelial cells. INSRR is considered as a tumor endothelial marker and its overexpression can promote angiogenesis (Nowak-Sliwinska et al., 2019). Recombinant ANXA2 can reduce endothelial permeability under hypoxic and flammatory factor injury states, indicating that ANXA2 may be involved in the maintenance of endothelial cell tightness (Li et al., 2019). In staining human cervical sections, strong expression of DUSP3 was found in endothelial cells, and experiments also demonstrated that DUSP3 is necessary for basic fibroblast growth factor induced microvascular growth (Amand et al., 2014). HBA1 expression in endothelial cells has been shown to control vascular tone and function (Sangwung et al., 2017). PGK1 is also thought to reduce atherogenesis (Zhang et al., 2020). What’s more, VTN is considered critical for thrombus formation in the setting of vascular injury (Bowley et al., 2017). GPI is enriched in the microvascular endothelial cells of synovial tissue from rheumatoid arthritis patients under hypoxic environment, and regulates the secretion of vascular endothelial growth factor from rheumatoid arthritis synovial fibroblasts to induce angiogenesis (Lu et al., 2017). In addition, the study of differentially expressed proteins related to varicocele mediated infertility showed that Nrf2 was an upstream regulator of ANXA2 (Panner Selvam et al., 2021). Inhibition of PGK1 can activate Keap1/Nrf2 pathway and stimulate cell protective antioxidant response (Bollong et al., 2018). The two proteins may be the potential targets of quercetin through Keap1/Nrf2 pathway. Further study can verify the role of these proteins on the effect of quercetin on H/R-HBMECs.

Admittedly, quercetin can exert protective effects on endothelial cells. But the specific targets of action still require further investigation, and whether there are additional pathways of action remains unknown.

In conclusion, this study showed that quercetin maintained the integrity of the blood-brain barrier by protecting endothelial cells. At the molecular level, quercetin may play a role in protecting endothelial cells by protecting against oxidative stress through the Keap1/Nrf2 pathway and inhibiting endoplasmic reticulum stress through the ATF6/GRP78 pathway. This study lays the foundation for TCM to treat CSVD by protecting endothelial cells.

## Data Availability

The datasets presented in this study can be found in online repositories. The names of the repository/repositories and accession number(s) can be found in the article/supplementary material.
